# 

**Published:** 1915-06

**Authors:** 


### The Seventh Pan-American Congress.

The seventh Pan-American Congress will meet in San Francisco, June 17-21, inclusive. It assembles pursuant to invitation of the President of the United States issued in accordance with an act of Congress approved March 3, 1915.

The countries and colonies embraced in the Congress are the Argentine Republic, Bolivia, Brazil, Canada, Colombia, Cuba, Chile, Costa Rica, El Salvador, Ecuador, Guatamala, Honduras, Haiti, Hawaii, Mexico, Martinique, Nicaragua, Panama, Paraguay, Peru, Santo Domingo, United States, Uruguay, Venezuela, British Guiana, Dutch Guiana, French Guiana, Jamaica, Barbadoes, St. Thomas and St. Vincent. The organization of the Congress is perfected in these countries and the majority of them have signified their intention to be represented by duly accredited delegates.

The Congress will meet in seven sections, viz.: (1) Medicine; (2) Surgery; (3) Obstetrics and Gynecology; (4) Anatomy, Physiology, Pathology and Bacteriology; (5) Tropical Medicine and General Sanitation; (6) Laryngology, Rhinology and Otology; (7) Medical Literature.

All members of the organized medical profession of the constituent countries are eligible and are invited to become members. The membership fee is $5.00 and entitles the holder to a complete set of the transactions. Advance registrations are solicited and should be sent with membership fee to the Treasurer, Dr. Henry P. Newman, Timken Building, San Diego, California.

### Deaths.

Dr. J. A. White, well known physician, died very suddenly at his home in Clarksville, April 16.

Dr. Walker, formerly of Hightower Valley, died at Weatherford April 22.

Dr. Thomas J. Murray died at his home in Glen Rose May 1, 1915.

Dr. A. M. Anderson died at the home of his brother in Olney after a lingering illness.

Dr. Jno. W. Nixon of Loco was drowned while returning from a call. The body was recovered some miles down the creek.

